# The Synergistic Effect Accelerates the Oxygen Reduction/Evolution Reaction in a Zn-Air Battery

**DOI:** 10.3389/fchem.2019.00524

**Published:** 2019-07-23

**Authors:** Yidan Zhang, Youmin Guo, Tao Liu, Fuxu Feng, Chunchang Wang, Haibo Hu, Mingzai Wu, Meng Ni, Zongping Shao

**Affiliations:** ^1^School of Physics and Materials Science, Anhui University, Hefei, China; ^2^Department of Building and Real Estate, The Hong Kong Polytechnic University, Hong Kong, China; ^3^State Key Laboratory of Materials-Oriented Chemical Engineering, College of Chemistry and Chemical Engineering, Nanjing University of Technology, Nanjing, China; ^4^Department of Chemical Engineering, Curtin University, Perth, WA, Australia

**Keywords:** Sr-doped Sm cobaltite perovskite, oxygen reduction reaction, oxygen evolution reaction, silver glue, synergistic effect

## Abstract

Perovskite oxides are promising electrocatalysts toward oxygen reduction reaction (ORR) and oxygen evolution reaction (OER) due to their abundance and high intrinsic catalytic activity. Here we introduce Ag into Sm_0.5_Sr_0.5_CoO_3−δ_ (SSC) to form a Ag-SSC catalyst by ultrasonication and apply it as the air electrode for a Zn-air battery. It finds that the introduction of Ag into SSC can transform the Ag-SSC into a good bifunctional electrocatalyst toward ORR as well as OER. For instance, a more active half-wave potential with a value of 0.76 V for ORR is obtained at 1,600 rpm, while the OER overpotential is 0.43 V at I = 10 mA cm^−2^. Further characterization demonstrates that the improved catalyst activity of the Ag-SSC can be assigned to the synergistic effect generated between the Ag and SSC phases. The Zn-air battery with the Ag-SSC as an electrode not only gives a same discharge-charge voltage gap (1.33 V) with that of commercial Pt/C (1.33 V) but also presents an equivalent current efficiency (45.7% for Ag-SSC and 45.3% for Pt/C) at 10 mA cm^−2^. Moreover, the stability for 110 cycles is better. This result indicates that the Ag-SSC catalyst shows promise for use as a bifunctional electrocatalyst toward OER and ORR.

## Introduction

The reactions toward the oxygen reduction and oxygen evolution (ORR and OER) play a great role in many conversion systems and energy storage including fuel cells (Xin et al., 2013), Zn-air batteries (Armand and Tarascon, 2008; Chen et al., 2015; Bu et al., 2017), solar fuel synthesis (Gray, 2009), and hydrogen production from water (Walter et al., 2010; Amano et al., 2018; Bu et al., 2019a). Up to now, the commercialization of these renewable energy technologies has been hindered by their sluggish dynamics, so it is urgent to exploit a bifunctional electrocatalyst with superior ORR/OER efficiency and long-term stability. Materials based on noble metals (e.g., Pt, Au) and their alloys (e.g., IrO_2_, RuO_2_, Pt-Au alloy) are well-known as effective oxygen catalysts for ORR/OER due to their electrocatalytic activities (Chen et al., 2015). However, their large-scale applications are seriously hampered because of their low storage, high cost, and durability (Hong et al., 2015; Zhang et al., 2016). Therefore, researchers have made great efforts to cut down the precious metal loading in noble metal-based catalysts via the use of alloys or a carbon support. However, the catalytic activities have been simultaneously offset while the noble metal loadings have been decreased. Therefore, it is indispensable to develop bifunctional electrocatalysts that are highly active and stable without precious metals to accelerate their practical application.

Perovskite oxide has been considered as one of the most hopeful alternatives to high efficiency ORR/OER electrocatalysts owing to an extraordinary variability of composition, fascinating physical and chemical properties, low cost and the fact that it is environmentally benign. However, the direct use of pure perovskites as ORR/OER catalysts is disadvantageous. For instance, the performance is limited by poor electronic conductivity at room temperature (Chen et al., 2015; Gupta et al., 2016) because electrocatalysis requires electrons to flow efficiently through the electrodes to produce high currents (Lee et al., 2015; Gupta et al., 2016). Thus, it is crucial to make up for this defect to improve the catalytic efficiency. Nowadays, It has been reported several strategies to improve the catalytic ability of perovskite oxides for ORR/OER, such as defect introduction (Wang et al., 2017), surface modification (Lin et al., 2019), nanostructure optimization (Jung et al., 2016), as well as composite material preparation (Xu et al., 2015; Zhao et al., 2017; Bu et al., 2019b). For example, Zhang et al. indicated that substituting Mg or Fe into the B-site of perovskite with the formation of LaNi_0.85_Mg_0.15_O_3_ and LaNi_0.8_Fe_0.2_O_3_ could enhance the ORR/OER activity of LaNiO_3_ (Du et al., 2014; Zhang et al., 2015). Zhou and Sunarso proved that the cubic LaNiO_3_ perovskite showed higher ORR/OER activity by quenching LaNiO_3_ oxide at different temperatures (Zhou and Sunarso, 2013). Jung et al. compounded a La-doped Ba_0.5_Sr_0.5_Co_x_Fe_1−x_O_3−δ_ nanosized particle of 50 nm exhibiting good excellent ORR/OER performance (Jung et al., 2016). To address the issue of low electronic conductivity, some reports tried to add precious metal elements into perovskite oxides, such as platinum (Ho et al., 2011). For instance, Chen et al. compounded ~1 nm Pt non-structural particles onto ~60 nm sized CaMnO_3_ nanoparticles. The synthesized materials showed better performance than Pt/C and CaMnO_3_ alone as a bifunctional electrocatalyst (Han et al., 2014). However, the manufacturing process was very complicated. Therefore, Zhu et al. proposed a simple ultrasonic technique to mix Pt/C and BSCF for obtaining an excellent bifunctional electrocatalyst through the synergistic effect between Pt/C and the BSCF perovskite (Zhu et al., 2014), and the feasibility of this method provides a good idea for our subsequent experiments.

Due to its reasonably high catalytic capacity in alkaline solutions for oxygen reduction and its low cost, Ag has been considered as a competitive ORR catalyst (Park et al., 2015). Therefore, mixing Ag into perovskite oxides for use as electrocatalysts will be a prospective strategy for promoting the catalytic ability of perovskite oxides as well as for lowering the cost of catalysts. As a kind of cathode candidate for a solid oxide fuel cell, Sm_0.5_Sr_0.5_CoO_3−δ_ (SSC) perovskite oxide has shown excellent oxygen reduction ability (Duan et al., 2018) and has also been demonstrated to be a promising electrocatalyst toward ORR and OER by adding Vulcan XC-72R or N-doped graphene as an electron-conducting phase (Velraj and Zhu, 2013; Bu et al., 2018). Herein, we designed a new type of catalyst slurry for a Zn-air battery by directly adding commercial silver glue into the SSC perovskite powder catalyst by ultrasonication. The composite slurry (Ag-SSC) exhibited much better ORR/OER activities than either Ag glue or SSC alone in alkaline electrolytes. This research gives prominence to the application of coupled perovskite oxide/silver glue for ORR/OER in a Zn-air battery and suggests the generation of a synergistic effect between the silver glue and the SSC.

## Materials and Methods

We used a sol-gel method that combined EDTA-citrate complexing to synthesize Sm_0.5_Sr_0.5_CoO_3−δ_ (SSC), and the relevant nitrates were used as the raw materials. First, we completely dissolved these raw materials in the required stoichiometric ratio with deionized water. Next, we introduced citric acid and EDTA as complexing agents and controlled the mixed solution pH at ~8 under the action of ammonia water. After heating the evaporated water, we obtained a transparent black gel. Finally, the gel was pre-fired at 240°C to form a precursor, and the final powder sample was obtained by calcining the precursor in air at 1,000°C.

A mixed homogeneous catalyst ink composite of SSC powders, conductive carbon (Super P Li), Nafion solution (5 wt %) and ethanol was prepared via ultrasonication. The SSC powder and conductive carbon was mixed at a ratio of 1:1, and the Nafion solution and ethanol were mixed at a ratio of 1:10. Another catalyst ink containing the above prepared SSC ink and an appropriate amount of silver glue was sonicated and labeled as Ag-SSC (the mass ratio of Ag was 25%). Before adding the prepared SSC ink, the silver glue was ground in ethanol solution for half an hour. Then, catalyst inks (5 μL) were drop cast onto a polished glassy carbon electrode (GC, 0.196 cm^2^, Pine Research Instrumentation) and dried in air for 2 h at room temperature. To ensure the cleanness of the GC electrode, we pre-polished the GC electrode using a polishing cloth with a 50 nm α-Al_2_O_3_ slurry followed by sonication in ethanol for 5 min and then rinsing with deionized water. In this work, the SSC catalyst has a loading amount of 0.232 mg_ox_ cm^−2^, except for the 20 wt% Pt/C and IrO_2_ catalysts (their loading amounts are 0.116 mg cm^−2^) used as reference catalyst inks.

We used a three-electrode cell (Pine Research Instrumentation) with a rotating disk electrode (RDE) measurement to execute electrochemical measurements using a potentiostat (CHI 650E). Ag/AgCl was applied as a reference electrode, platinum wire was utilized as a counter electrode and KOH aqueous solution (0.1 M) was utilized as the electrolyte. Before each test, O_2_ was introduced into the KOH solution to obtain an O_2_ saturated solution, ensuring the whole test was conducted under an O_2_ atmosphere. We recorded cyclic voltammetry (CV) curves for the catalysts between 0.2 and −0.8 V until a stable curve was obtained. The scan rate was 100 mV s^−1^. We recorded linear sweeping voltammetry (LSV) curves for the ORR tests via use of the RDE at various rotation speeds (2,000, 1,600, 1,200, 800, and 400 rpm) between 0.2 and −0.8 V. The scan rate was set as 5 mV s^−1^. Both CV and LSV were scanned in the negative mode. We investigated the accelerated stability of catalysts at 1,600 rpm for one thousand cycles through a potential cycle. The scan rate was 100 mV s^−1^, and the potential was changed from 0.2 to −0.8 V. During the reaction for oxygen evolution, LSV curves for the catalysts were recorded. The scan rate was 5 mV s^−1^, and the potential was scanned in positive mode from 0.2 to 0.1 V. In this study, we converted all potentials from vs. Ag/AgCl to the reversible hydrogen electrode (RHE) scale.

The electrochemical test on a zinc-air battery was conducted at room temperature with Zn plate as an anode. A 1 cm^2^ catalyst coated carbon paper was applied as an air electrode, while a 6 M KOH solution containing 0.2 M Zn(Ac)_2_ was utilized as an electrolyte. Before the test, the prepared Ag-SSC catalyst slurry was drop cast onto carbon paper with an amount of 1 mg cm^−2^.

The crystallization of the SSC powder was measured by X-ray powder diffraction (XRD, Bruker AXS D8 Advance), which scanned from 20 to 80° with Cu Kα radiation. The microstructure of the catalysts was characterized by scanning electron microscopy (SEM, Hitachi S-4800) equipped with X-ray energy dispersive spectroscopy (EDX), with the accelerating voltage set as 5 kV. The specific surface area of the samples was measured by the Brunauer-Emmett-Teller (BET) method using nitrogen adsorption and desorption isotherms on a Micromeritics ASAP 2020 system. X-ray photoelectron spectroscopy (XPS, Thermo-Fisher ESCALAB 250Xi), which scanned with a Mg Kα achromatic X-ray source, was used to identify the chemical compositions and surface element states of the samples.

## Result and Discussion

**Figure 1:**
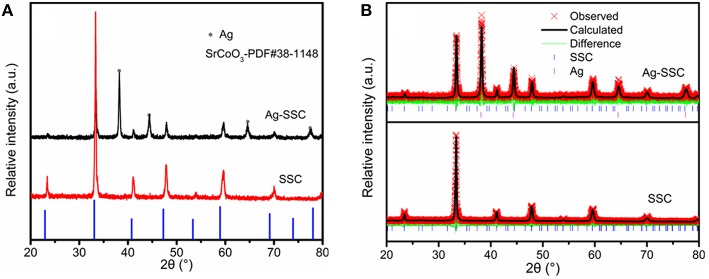
XRD patterns **(A)** and Rietveld refinement results **(B)** of the SSC and Ag-SSC.

The Ag-SSC composite as well as SSC were characterized by XRD scanning from 20 to 80°. As presented in Figure 1, sharp and intense diffraction peaks suggested that a well-crystallized phase of SSC was synthesized at 1,000°C. The characteristic reflections for the SSC were observed at 2θ = 23.4, 33.33, 41.08, 47.91, 53.89, 59.65, and 69.99° corresponding to the lattice planes of (100), (110), (111), (200), (210), (211), and (220), respectively. All the peaks shifted to higher 2θ values compared with SrCoO_3_ (JCPDS file no. 38-1148), indicating that a fraction of the larger Sr^2+^ ions (1.12 Å) was replaced by smaller Sm^3+^ ions (0.96 Å). The XRD patterns for SSC and Ag-SSC were further analyzed by Rietveld refinement. The results showed that the synthesized SSC had a space group of I4/mmm. The values for *a, b*, and *c* were found to be 7.605, 7.605, and 15.195 Å, respectively. The corresponding factor of R_b_ was 2.45%, confirming the high reliability of the refinement results. After mixing the silver glue, the resulting Ag-SSC composite showed four new diffraction peaks due to Ag, which proved the existence of silver.

**Figure 2:**
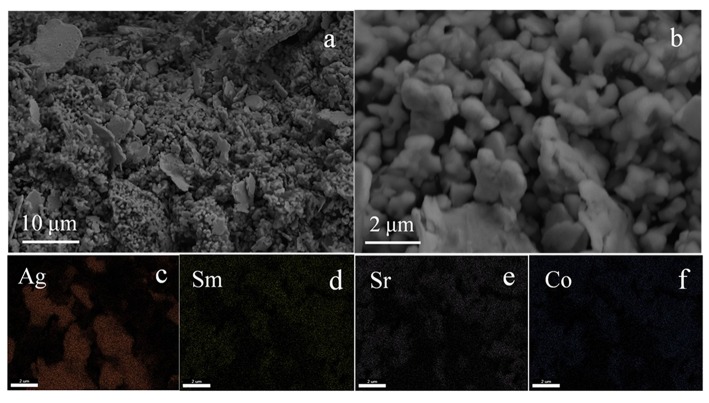
SEM images of Ag-SSC composite **(a,b)** and the corresponding element mappings of Ag **(c)**, Sm **(d)**, Sr **(e)**, Co **(f)**.

The SEM morphology of the Ag-SSC catalyst is displayed in Figure 2. The Ag particle was fully distributed into the SSC nanoparticles with sizes of 1~10 μm (Figure 2a). However, the Ag particle had an irregular shape (Figure 2a), and the distribution of Ag in the SSC particles was demonstrated to be uneven (Figure 2c), which will adversely affect the activity of the composite catalyst (Guo et al., 2010; Park et al., 2015; Wang et al., 2018). In addition, the content of Ag in Ag-SSC was calculated based on the X-ray energy dispersive spectroscopy (EDX) results and listed in Table 1. The content of Ag in Ag-SSC was approximately 25.21 wt % ± 1.83%, which was in line with the ideal design value of 25% in general. The Brunauer-Emmett-Teller (BET) surface area of the SSC was 6.452 m^2^g^−1^ calculated from N_2_ isotherms at 77 K.

**Table 1:** The X-ray energy dispersive spectroscopy (EDX) results of Ag-SSC.

**Element**	**Weight %**	**Atomic %**	**Error %**
Ag	25.21	6.90	1.83
Sm	16.05	3.13	4.58
Sr	7.85	2.76	4.05
Co	11.79	5.94	3.87
Others	39.10	81.27	7.89

**Figure 3:**
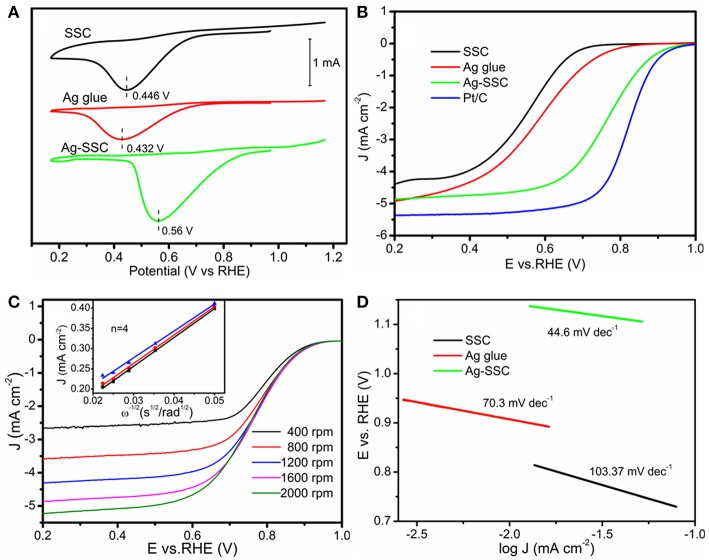
ORR CV curves of SSC, Ag glue, and Ag-SSC catalyst in an O_2_-saturated 0.1 M KOH solution at a scan rate of 100 mV s^−1^ **(A)**. LSV curves of SSC, Ag glue, and Ag-SSC catalyst in an O_2_-saturated 0.1 M KOH solution at a scan rate of 5 mV s^−1^ at 1,600 rpm **(B)**. LSV curves of Ag-SSC catalyst in an O_2_-saturated 0.1 M KOH solution at a scan rate of 5 mV s^−1^ at 400~2,000 rpm and K–L plots for Ag-SSC at 0.55, 0.60, and 0.65 V **(C)**. Tafel plots of SSC, Ag glue, and Ag-SSC **(D)**.

Figure 3A shows the cyclic voltammetry (CV) tests for SSC, Ag glue, and Ag-SSC catalysts. The peak voltages for SSC, Ag glue, and Ag-SSC were 0.446, 0.432, and 0.560 V, respectively. Obviously, the Ag-SSC exhibited the highest peak voltage among these catalysts, which proved an increased electrocatalytic capacity for ORR due to the addition of Ag glue. To prove this view, we also measured the linear sweeping voltammetry (LSV) curves for all three catalysts. As demonstrated in Figure 3B, Ag-SSC presented not only a more positive half-wave potential at 0.76 V but also a higher limited I = 4.87 mA cm^−2^ at 1,600 rpm than that of Ag glue alone and SSC alone (0.53 V/4.92 mA cm^−2^ for Ag glue and 0.55 V/4.39 mA cm^−2^ for SSC), which was similar to that obtained for the benchmark Pt/C catalyst (0.83 V/5.37 mA cm^−2^). This result further indicated that the addition of Ag improved the catalytic ability of SSC for ORR.

We further used the Koutecky-Levich equation to evaluate the ORR kinetics of various catalysts. Through Equation (1), the electron transfer number (n) during the ORR can be calculated (Xiong et al., 2010; Wang et al., 2016):

(1) $$\frac{1}{J}=\frac{1}{J_{K}}+\frac{1}{J_{L}}=\frac{1}{nFkC^{0}}+\frac{1}{0.62nFD_{O_{2}}^{\;2/3}v^{-1/6}C^{0}w^{1/2}}$$

where *w* is the electrode rotating rate; *v* is the kinematic viscosity of the electrolyte via 0.1 M KOH (0.01 cm^2^ s^−1^); ***J***, ***J***_***K***_ and ***J***_***L***_ are, respectively, the measured current density, kinetic density and diffusion-limiting current density; ***F*** is the Faradic constant, 96,485 C mol^−1^; $D_{O_{2}}^{\;}$ represents the oxygen diffusion coefficient via 0.1 M KOH solution (1.9 × 10^−5^ cm^2^ s^−1^); ***C***^**0**^ represents the oxygen bulk concentration (1.1 × 10^−6^ mol cm^−3^); and ***k*** represents the electron transfer rate constant. From the K-L plots for Ag-SSC fixed at 0.55, 0.60, and 0.65 V (Figure 3C), the data show that the transferred electron number approached 4. This result effectively indicated that the ORR process was almost carried out by a four-electron path under the action of the Ag-SSC catalyst in the alkaline electrolyte, suggesting that an advantageous one-step four-electron ORR process with the formation of H_2_O directly occurred for the Ag-SSC catalyst according to the following equation:

(2) $$O_{2}+4e^{-}+4H^{+}\to 2H_{2}O$$

In fact, there is another reaction pathway in the ORR process: O_2_ first incurs a two-electron reduction to H_2_O_2_ and then continues to an extra two-electron reduction to H_2_O. This reaction can be described as follows (Ahmed et al., 2011):

(3) $$O_{2}+2e^{-}+2H^{+}\to H_{2}O_{2}$$

(4) $$H_{2}O_{2}+2e^{-}+2H^{+}\to 2H_{2}O$$

To further obtain kinetic information for the ORR, we recorded the Tafel plots for the different catalysts. From Figure 3D, the Tafel plots for SSC, Ag glue, and Ag-SSC were 103.37, 70.30, and 44.60 mV dec^−1^, respectively. These results demonstrated that Ag-SSC had an exceptional ORR activity. The reason for the enhancement for the Ag-SSC catalyst will be discussed later.

**Figure 4:**
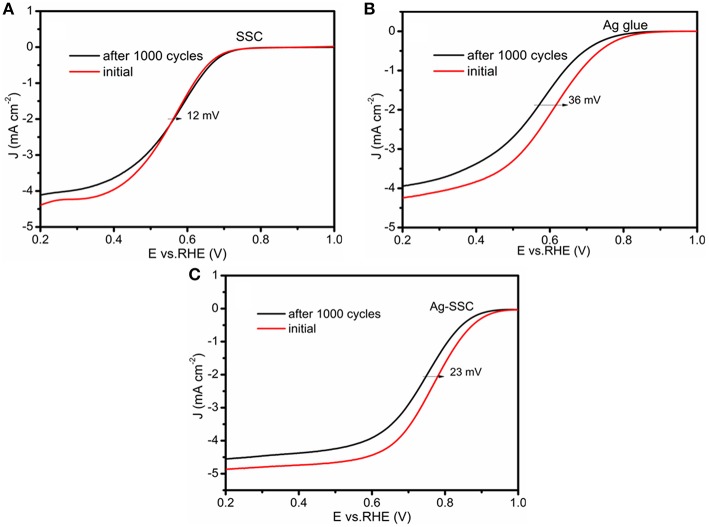
ORR LSV curves of SSC **(A)**, Ag glue **(B)**, and Ag-SSC **(C)** at 1,600 rpm at a scan rate of 100 mV s^−1^ in an O_2_-saturated 0.1 M KOH solution before and after accelerated aging test.

The cycle stability is an important performance indicator that determines whether an electrocatalyst is practical. Herein, the stability of the SSC, Ag glue and Ag-SSC were verified by an accelerated aging test. As shown in Figure 4, the three catalyst slurries were subjected to 1,000 continuous potential cycles for obtaining a new LSV curve. The experimental settings were the same as that used in the above investigation. The obtained half-wave potential losses of the SSC, Ag glue, and Ag-SSC were ~12, ~ 36, and ~23 mV, respectively. This obviously showed that the stability of Ag glue alone was bad, similar to the stability of the Pt/C catalyst (Zhu et al., 2015). Even if the SSC catalyst showed the best stability, its catalytic activity was too poor for practical application. After mixing Ag glue into the SSC oxide, the stability of the Ag-SSC composite was much higher than that of Ag glue and the catalytic activity was enhanced substantially. This result indicated that introducing Ag glue into SSC oxide could improve its catalytic activity as well as the stability.

**Figure 5:**
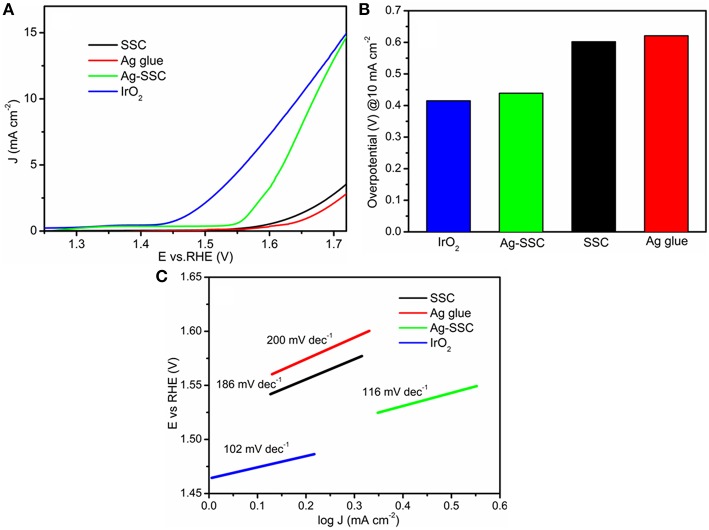
OER LSV curves of SSC, Ag glue, Ag-SSC and IrO_2_ at 1,600 rpm at a scan rate of 100 mV s^−1^ in an O_2_-saturated 0.1 M KOH solution **(A)**. Overpotentials of SSC, Ag glue, Ag-SSC, and IrO_2_ at a current density of 10 mA cm^−2^ **(B)** and Tafel plots of SSC, Ag glue, and Ag-SSC and IrO_2_ **(C)**.

To discover whether the catalyst has bifunctional performance or not, we further tested the OER activity by RDE. Figure 5A displays the LSV curves for the OER on SSC, Ag glue, Ag-SSC, and IrO_2_ at 1,600 rpm. As expected, the Ag-SSC catalyst showed better OER performance than either the Ag glue or SSC catalyst alone. For instance, the onset potentials for SSC, Ag glue, and Ag-SSC were 1.59, 1.58, and 1.54 V, respectively. Meanwhile, Ag-SSC showed a current density that was very close to that obtained for IrO_2_. The overpotential of the Ag-SSC catalyst was 0.43 V when the current density was 10 mA cm^−2^, which was smaller than the overpotential of SSC (0.60 V) and Ag glue (0.62 V) alone and approached that of IrO_2_ (0.40 V) (Figure 5B). This result proved that Ag-SSC also had an active OER activity. In addition, the Tafel slopes were calculated to obtain more kinetic information for the OER and are given in Figure 5C. The Tafel slopes for SSC, Ag glue, Ag-SSC and IrO_2_ were 186, 200, 116, and 102 mV dec^−1^, respectively. Moreover, the potential difference (ΔE) was calculated at a constant OER current of 10 mA cm^−2^ and ORR current of −3 mA cm^−2^, as shown in Table 2. The ΔE values were 1.33, 1.31, and 0.93 V for SSC, Ag gluem, and Ag-SSC, respectively. This result obviously demonstrated that ΔE was decreased when Ag was introduced into the SSC perovskite oxide.

**Table 2:** Oxygen electrode activities of SSC, Ag glue and Ag-SSC.

	**ORR@-3 mA cm^**−2**^**	**OER@10 mA cm^**−2**^**	**ΔE**
SSC	0.50 V	1.83 V	1.33 V
Ag glue	0.54 V	1.85 V	1.31 V
Ag-SSC	0.73 V	1.66 V	0.93 V

**Figure 6:**
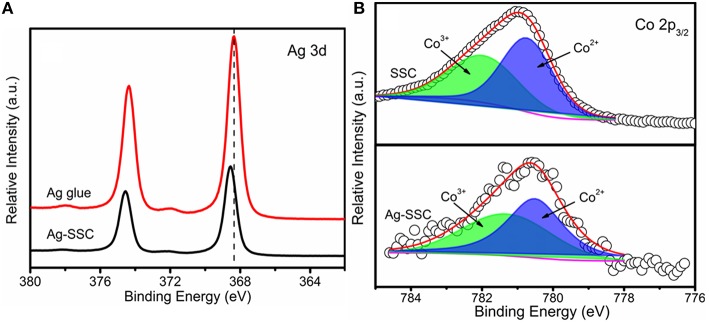
XPS spectra of Ag 3d **(A)** and Co 2p_3/2_ **(B)**.

**Figure 7:**
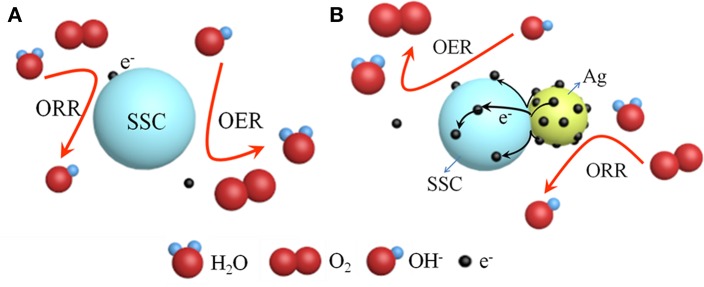
Schematic representation of SSC **(A)** and electron transfer in Ag-SSC composite **(B)** for ORR/OER.

According to the above electrochemical results, we discovered that the Ag-SSC composite catalyst had higher OER and ORR catalytic ability than the one-component Ag and SSC perovskite oxide, which indicated that there was a certain synergistic effect at play in the Ag-SSC composite (Zhu et al., 2014). Therefore, the chemical states for Ag and Co in the Ag-SSC composite were detected by XPS. Both Ag glue alone and the Ag-SSC composite exhibited two major peaks with binding energy values at 374.35, 368.35 eV and 374.55, 368.55 eV, respectively (Figure 6A). The spin energy difference in these data was 6 eV, which was consistent with the reported data (Zhang et al., 2016). However, the Ag 3d peak for Ag-SSC showed a slight shift to higher binding energy, indicating that interaction of the perovskite oxide with Ag leads to the transfer of electrons from Ag to SSC (Hong et al., 2015). The Co 2p_3/2_ core spectra for SSC and Ag-SSC are shown in Figure 6B. From the fitted curve, the valence of Co in SSC was determined to be between +2 and +3, and the Co^2+^ area ratio was 54%, while in the Ag-SSC structure, the average Co value was close to +2, and the Co^2+^ area ratio was 66%, which was lower than that of Co in the SSC, which indicated that the electronic structure of Co in the Ag-SSC composite was changed due to the action of the Ag (Bu et al., 2018). Figure 7 presents a schematic for the electron transfer in the Ag-SSC composite. We consider that the surface electronic transfer in the Ag-SSC composite was optimized by adding Ag into the SSC perovskite oxide, thereby leading to an increased ORR activity. A similar effect of the electron transfer effect has also been previously reported in other systems (Liu and Mustain, 2013; Han et al., 2014; Wang et al., 2018).

**Figure 8:**
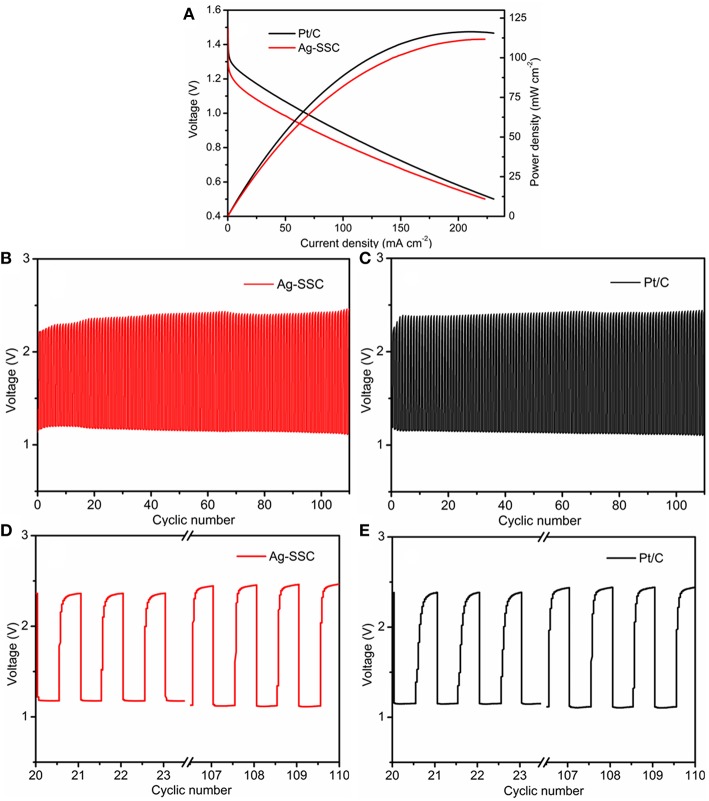
Discharge polarization curve and power density of rechargeable Zn–air battery **(A)**. Galvanostatic discharge-charge cycling curves for Ag-SSC **(B)** and Pt/C **(C)** at 10 mA cm^−2^ at different cycles, and comparison of the before and after cycles for Ag-SSC **(D)** and Pt/C **(E)**.

To demonstrate the practical use of the Ag-SSC composite, rechargeable Zn-air batteries were assembled with Ag-SSC applied as an air electrode material. Figure 8A shows the discharge polarization curves and power densities for our laboratory-made Zn-air battery. The discharge voltage of the Ag-SSC cathode in the battery was 0.98 V at a current density of 50 mA cm^−2^ during the discharge process, which was more inferior to that of a Pt/C cathode (1.06 V) in the same test condition. The peak power density for the Ag-SSC reached 104.46 mW cm^−2^ at 0.65 V, which was slightly smaller than the density of a commercial Pt/C catalyst (113.88 mW cm^−2^). Based on these data, we consider that the activity of Ag-SSC was slightly inferior to that of the Pt/C electrode, although it showed promising catalytic activity toward OER and ORR. The possible reason for this phenomenon is the uneven distribution of Ag glue in the SSC, resulting in an inability to perform a complete electron transfer between the two phases, thereby reducing the synergistic efficiency.

As we know, for rechargeable metal-air batteries, superior stability for the catalyst is an indispensable condition for an oxygen catalyst; therefore, the operation stability of Ag-SSC for the air electrode of a Zn-air battery was investigated (Zhao et al., 2018). Figures 8B–E shows the galvanostatic discharge-charge cycling curves at I = 10 mA cm^−2^ for the Zn-air battery with each cycle being 20 min. It could be seen that under the same test conditions, the Ag-SSC catalyst as a cathode material had a stability property that was similar to that found for the Pt/C catalyst. At the beginning of the loop test, the charge and discharge potentials for the Ag-SSC cathode were 2.35 and 1.18 V, respectively (Figure 8D), and a high voltaic efficiency of 50.2% was obtained, which exceeded that found for the commercial Pt/C catalyst (2.37/1.15 V, 48.5%) (Figure 8E). After 110 cycles, the charge and discharge potentials for Ag-SSC were 2.45/1.12 V and the corresponding values for Pt/C were 2.43/1.10 V. The high voltaic efficiency for Ag-SSC was reduced to 45.7%, while that for Pt/C was decreased to 45.3%. These results revealed that the Ag-SSC cathode had a better long-term cycling stability, even better than a commercial Pt/C cathode. Thus, we conclude that the Ag-SSC composite is an effective catalyst material for use in a rechargeable Zn-air battery.

## Conclusion

In this study, we reported a promising air electrode for a Zn-air battery prepared through simple ultrasonic mixing of Ag glue with a Sm_0.5_Sr_0.5_CoO_3−δ_ (SSC) perovskite oxide. This composite catalyst slurry was demonstrated to show better ORR performance and OER performance than either the SSC or Ag glue alone, which indicates the existence of a synergistic effect between the Ag and SSC phases. According to XPS spectra, we believe that there is electron transfer between the Ag and Co elements. Finally, using the Ag-SSC composite material as an air electrode, a rechargeable Zn-air battery was assembled, which showed a discharge-charge voltage gap similar to that obtained for a Zn-air battery using commercial Pt/C as the air electrode (1.33 V, 1.32 V for Pt/C). In addition, our assembled Zn-air battery showed a high current efficiency (45.7%, 45.3% for Pt/C) at 10 mA cm^−2^, as well as a better cycle stability after 110 cycles. Finally, these experiments showed that our designed bifunctional electrocatalyst has good prospects for application in Zn-air batteries.

## Data Availability

All datasets generated for this study are included in the manuscript and/or the supplementary material.

## Author Contributions

YG contributed conception and design of the manuscript. YZ conducted an experiment and wrote the first draft of the manuscript. All authors contributed to manuscript revision, read, and approved the submitted version.

### Conflict of Interest Statement

The authors declare that the research was conducted in the absence of any commercial or financial relationships that could be construed as a potential conflict of interest.
